# Low admission pulse pressure and increased in-hospital mortality in patients with heart failure

**DOI:** 10.3389/fcvm.2026.1747168

**Published:** 2026-04-30

**Authors:** Liying Zhong, Meng Wei, Xianhui Zhou

**Affiliations:** Department of Cardiac Pacing and Electrophysiology, The First Affiliated Hospital of Xinjiang Medical University, Urumqi, China

**Keywords:** heart failure, in-hospital mortality, mid-range ejection fraction, preserved ejection fraction, pulse pressure, reduced ejection fraction

## Abstract

**Background:**

Pulse pressure (PP) can predict out-of-hospital cardiovascular events in heart failure (HF), yet its prognostic value for in-hospital mortality remains incompletely elucidated. This study aims to investigate the independent association between admission PP and the risk of in-hospital mortality, as well as its consistency across different left-ventricular ejection fraction (LVEF) phenotypes.

**Methods:**

We retrospectively analyzed clinical data from patients with heart failure admitted to the First Affiliated Hospital of Xinjiang Medical University between March 2012 and September 2023 via the electronic medical record system. A random-forest algorithm ranked predictor importance; variables among the top 15 and LVEF were carried forward to multivariable logistic regression to quantify the association between PP and (i) all-cause mortality and (ii) cardiac death. Restricted cubic splines modeled dose-response relationships. Analyses were repeated stratified by LVEF categories.

**Results:**

A total of 21,768 patients were included in the analysis, with 1,541 (7.1%) experiencing in-hospital mortality. After adjustment for the 16 covariates, PP <30 mmHg independently predicted both all-cause mortality (OR 1.31, 95% CI 1.06–1.60) and cardiac death (OR 1.80, 95% CI 1.38–2.35). Restricted cubic spline plots demonstrated that when PP was <50 mmHg, a lower PP was associated with a higher risk of in-hospital all-cause mortality and cardiac death. The relationship between PP and in-hospital mortality was consistent across LVEF strata.

**Conclusion:**

Low admission PP identifies hospitalized HF patients at heightened risk of in-hospital death, irrespective of LVEF. Integration of PP into early risk-stratification algorithms may facilitate rapid triage and intensified monitoring in this vulnerable cohort.

## Introduction

Heart failure is a multifactorial syndrome that arises from structural and/or functional derangements of the heart, resulting in impaired ventricular filling and/or ejection. It is phenotypically classified—according to left-ventricular ejection fraction—into heart failure with reduced ejection fraction (HFrEF, LVEF ≤40%), mildly reduced ejection fraction (HFmrEF, LVEF 41%–49%), and preserved ejection fraction (HFpEF, LVEF ≥50%). Although contemporary guideline-directed therapies have markedly improved long-term outcomes, in-hospital mortality remains unacceptably high and displays substantial regional and phenotypic heterogeneity. Heart failure has emerged as a significant healthcare challenge due to its escalating resource utilization and medical costs. In contrast to high-income countries, cardiovascular mortality remains the leading cause of death in China, with no evidence of reversal (1, 2). Early identification of high-risk populations with heart failure, reduction of in-hospital mortality, and improvement of patient prognosis can alleviate the burden of heart failure on patients, their families, and healthcare expenditures.

Pulse pressure, an easily accessible and cost-effective clinical parameter, serves as a key determinant in risk assessment for myocardial infarction, atrial fibrillation, stroke, and all-cause mortality (3–5). PP levels are directly related to stroke volume and arterial compliance, while being indirectly influenced by heart rate, peripheral vascular resistance, and cardiac output. Owing to the complex and distinct pathophysiological mechanisms across heart failure phenotypes with different ejection fractions (EF), inconsistencies persist in existing research regarding whether PP maintains stable predictive efficacy across these phenotypes and whether its prognostic implications are consistent or heterogeneous. Specifically, in HFrEF, low PP indicates an elevated risk of death (6, 7). In HFpEF, earlier studies suggested that PP is not an independent determinant of HF-related mortality, or that this association is modified by systolic blood pressure (6, 8). In contrast, a recent report revealed a J-shaped relationship between PP and mortality, which is independent of systolic blood pressure levels (9). For HFmrEF, relevant investigations remain scarce, and the available findings are highly contradictory (7, 9).Despite the abundance of studies exploring the association between PP and HF-related mortality, most have focused on the risk of out-of-hospital death, with insufficient attention paid to its prognostic value for in-hospital mortality. Recent studies have demonstrated that admission PP predicts in-hospital mortality in acute type A aortic dissection (10) and may inform the haemodynamic response to inotropes—and thereby early death risk—in cardiogenic shock (11). Yet these observations derive from distinct acute cardiovascular entities and cannot be extrapolated to the heart failure population.

Therefore, the present study aims to leverage real-world data to investigate the impact of pulse pressure on in-hospital mortality in patients with heart failure and to determine whether there is heterogeneity in this effect across the three HF phenotypes stratified by ejection fraction.

## Methods

### Study population

**Figure 1:**
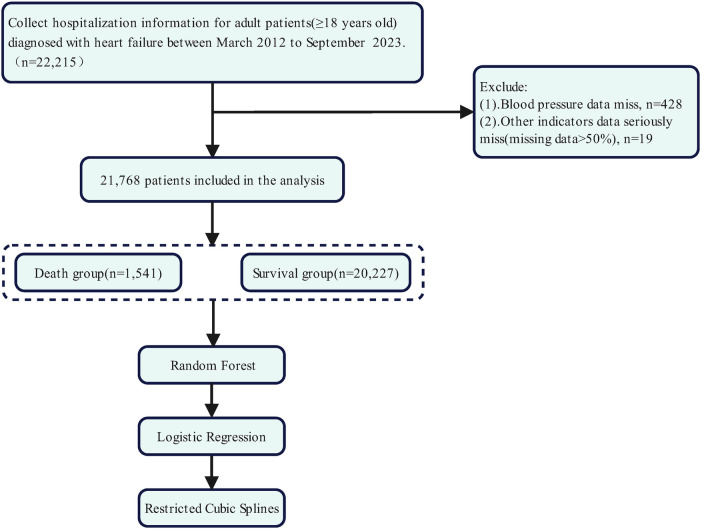
Study flow chart.

We retrospectively identified all consecutive adults aged ≥18 years who were admitted to the First Affiliated Hospital of Xinjiang Medical University between March 2012 and September 2023, with a primary or secondary diagnosis of heart failure documented in either their discharge summary or death record. After excluding 428 individuals with missing vital status or admission blood pressure and 19 with >50% missing covariates, 21,768 patients composed the analytic cohort (Figure 1). The study protocol was approved by the local ethics committee (K202403-48-2503A-Y1) and, given its retrospective nature and minimal-risk designation, patient consent was waived.

### Data ascertainment

Data were retrieved from the hospital's electronic medical record system, with a total of 53 clinical variables selected, including: (i) demographics; (ii) lifestyle factors and comorbid burden (hypertension, diabetes, coronary atherosclerotic heart disease, cerebral infarction); (iii) physical findings, admission vital signs—including pulse pressure calculated as systolic minus diastolic blood pressure measured using calibrated electronic sphygmomanometers. The first valid blood pressure reading obtained within 24 h of hospital admission was used for PP calculation; (iv)laboratory values, and echocardiographic metrics—including LVEF measured by the first transthoracic echocardiography after admission; and (v) pre-admission and in-hospital therapies (Supplementary Table S1).

Outcome adjudication followed prespecified definitions. The primary endpoint was in-hospital all-cause mortality, defined as death from any etiology during the index admission. Secondary endpoints were cardiac death, adjudicated by two independent cardiologists using explicit chart review and classified as death attributable to acute myocardial infarction, worsening HF, cardiogenic shock, malignant arrhythmia, or other structural/functional cardiac disorders.

### Statistical analysis

Normality of continuous variables was formally evaluated before selection of appropriate summary and inferential statistics. Non-normally distributed continuous variables were expressed as median (Q1, Q3) and compared across groups with the Kruskal–Wallis test; categorical variables are reported as *n* (%) and tested with *χ*² test. Pulse pressure, LVEF, and additional the top 15 predictors selected by a preliminary random-forest model were converted into dummy variables (details shown in Supplementary Table S2), with respective reference groups defined to quantify odds ratios for all-cause mortality and cardiac death.

A two-stage modeling approach was employed to identify independent predictors of in-hospital mortality. Univariable logistic regression was first performed to screen candidate predictors of in-hospital death. Variables exhibiting *P* < 0.05 were subsequently entered into multivariable logistic models constructed with bidirectional stepwise selection, with entry and exit thresholds set at *P* < 0.05. The variance inflation factor (VIF) was computed for all variables in the final model to assess multicollinearity, with a VIF <5 considered acceptable. To clarify whether the association between PP and in-hospital mortality differs across the three heart failure phenotypes stratified by EF, we examined the interaction between PP and EF. The statistical significance of this interaction was assessed using the likelihood ratio test. Furthermore, restricted cubic spline (RCS) plots were generated to depict the non-linear relationships of PP with in-hospital all-cause mortality and cardiac death with the number of knots set at 4 (5th, 35th, 65th, and 95th percentiles). This RCS analysis was conducted for the overall cohort and was subsequently stratified by EF phenotypes to graphically explore the consistency and shape of the association across distinct patient phenotypes.

All analyses were performed with R version 4.5.0 and SPSS version 26.0. Two-sided *P*-values <0.05 were considered statistically significant.

## Results

### Baseline characteristics

Among 21,768 in-patients with heart failure, 1,541 (7.08%) died during the index hospitalization. A random-forest model with death as the outcome and all baseline variables except pulse pressure ranked the following as the 15 most influential predictors: C-reactive protein (CRP), D-dimer, body mass index (BMI), NT-proBNP, age, white-blood-cell count (WBC), direct bilirubin (DBIL), free triiodothyronine (FT_3_), glucose, aspartate aminotransferase (AST), albumin, serum sodium, alanine aminotransferase (ALT), serum calcium and heart rate (HR) (Supplementary Figure S1).

For analytic clarity, participants were trichotomised by admission pulse pressure: low PP (<30 mmHg; *n* = 1,480), intermediate PP (30–60 mmHg; *n* = 14,960), and high PP (>60 mmHg; *n* = 5,328). Table 1 summarises baseline characteristics. Patients in the low-PP stratum were younger, had faster heart rates, and exhibited lower LVEF than those with PP ≥30 mmHg. Furthermore, among the three groups, the prevalence of hypertension, diabetes, coronary atherosclerotic heart disease, and cerebral infarction was lowest in the PP <30 mmHg group.Across the three groups, BMI, white-blood-cell count, transaminases, direct bilirubin, albumin, sodium, calcium, glucose, NT-proBNP, and free triiodothyronine differed significantly (all *P* < 0.05).

**Table 1:** Comparison of baseline characteristics according to pulse pressure categories.

Variables	Total (*n* = 21,768)	30 ≤ PP ≤60 mmHg (*n* = 14,960)	PP <30 mmHg (*n* = 1,480)	PP >60 mmHg (*n* = 5,328)	*P*
Age, years	63.16 (52.09, 73.37)	61.91 (51.03, 72.20)	56.87 (48.06, 67.03)	68.18 (57.65, 76.88)	<0.001
HR, times/minute	82.00 (76.00, 96.00)	82.00 (76.00, 95.00)	93.00 (80.00, 105.00)	82.00 (74.00, 93.00)	<0.001
BMI, kg/m^2^	25.15 (22.21, 28.69)	25.01 (22.04, 28.58)	25.10 (22.23, 28.65)	25.47 (22.78, 28.93)	<0.001
WBC, 10^9^/L	7.30 (5.80, 9.30)	7.30 (5.80, 9.34)	7.46 (5.95, 9.40)	7.24 (5.78, 9.18)	0.020
ALT, U/L	23.09 (14.96, 42.00)	23.98 (15.12, 42.91)	31.05 (18.41, 60.00)	20.00 (13.40, 34.00)	<0.001
AST, U/L	26.00 (18.55, 43.56)	26.14 (18.70, 44.60)	32.80 (22.10, 59.40)	24.10 (17.60, 37.49)	<0.001
DBIL, μmol/L	2.97 (0.30, 5.31)	3.05 (0.30, 5.40)	3.54 (0.30, 7.20)	2.62 (0.30, 4.80)	<0.001
ALB, g/L	36.70 (32.05, 40.70)	36.90 (32.20, 40.80)	36.28 (32.38, 40.11)	36.30 (31.60, 40.35)	<0.001
Na^+^, mmol/L	139.70 (136.66, 142.17)	139.60 (136.55, 142.00)	139.10 (136.22, 141.99)	140.00 (137.00, 142.60)	<0.001
Ca^2+^, mmol/L	2.16 (2.05, 2.27)	2.17 (2.06, 2.27)	2.16 (2.05, 2.26)	2.15 (2.04, 2.26)	<0.001
Glu, mmol/L	6.24 (4.98, 7.70)	6.19 (4.94, 7.64)	6.17 (4.95, 7.63)	6.39 (5.09, 7.91)	<0.001
CRP, mg/L	25.27 (16.30, 31.33)	25.36 (16.41, 31.35)	24.84 (15.10, 31.45)	25.20 (16.34, 31.27)	0.507
D-dimer, ng/mL	633.00 (214.00, 1,009.00)	632.00 (211.00, 1,003.25)	627.50 (214.75, 1,035.25)	639.00 (221.00, 1,015.25)	0.244
NT-proBNP, ng/L	2,962.00 (1,130.00, 5,166.00)	2,910.00 (1,104.00, 5,063.75)	3,410.00 (1,710.00, 5,852.50)	2,960.00 (1,053.75, 5,335.25)	<0.001
FT_3_, pmol/L	3.77 (2.96, 4.50)	3.78 (2.97, 4.52)	3.84 (2.99, 4.50)	3.72 (2.91, 4.43)	<0.001
LVEF, %	54.23 (42.79, 62.00)	54.00 (42.00, 62.00)	48.11 (38.92, 59.00)	56.48 (45.29, 62.14)	<0.001
All-cause mortality, *n* (%)					0.027
Survival	20,227 (92.92)	13,939 (93.18)	1,353 (91.42)	4,935 (92.62)	
Death	1,541 (7.08)	1,021 (6.82)	127 (8.58)	393 (7.38)	
Cardiac death, *n* (%)					<0.001
Survival	21,194 (97.36)	14,585 (97.49)	1,408 (95.14)	5,201 (97.62)	
Death	574 (2.64)	375 (2.51)	72 (4.86)	127 (2.38)	
HTN, *n* (%)					<0.001
No	9,601 (44.11)	7,998 (53.46)	894 (60.41)	709 (13.31)	
Yes	12,167 (55.89)	6,962 (46.54)	586 (39.59)	4,619 (86.69)	
DM, *n* (%)					<0.001
No	12,197 (56.03)	8,684 (58.05)	880 (59.46)	2,633 (49.42)	
Yes	9,571 (43.97)	6,276 (41.95)	600 (40.54)	2,695 (50.58)	
CAD, *n* (%)					<0.001
No	11,751 (53.98)	8,102 (54.16)	875 (59.12)	2,774 (52.06)	
Yes	10,017 (46.02)	6,858 (45.84)	605 (40.88)	2,554 (47.94)	
CI, *n* (%)					<0.001
No	20,062 (92.16)	13,850 (92.58)	1,396 (94.32)	4,816 (90.39)	
Yes	1,706 (7.84)	1,110 (7.42)	84 (5.68)	512 (9.61)	

### Determinants of in-hospital mortality in the overall cohort

**Figure 2:**
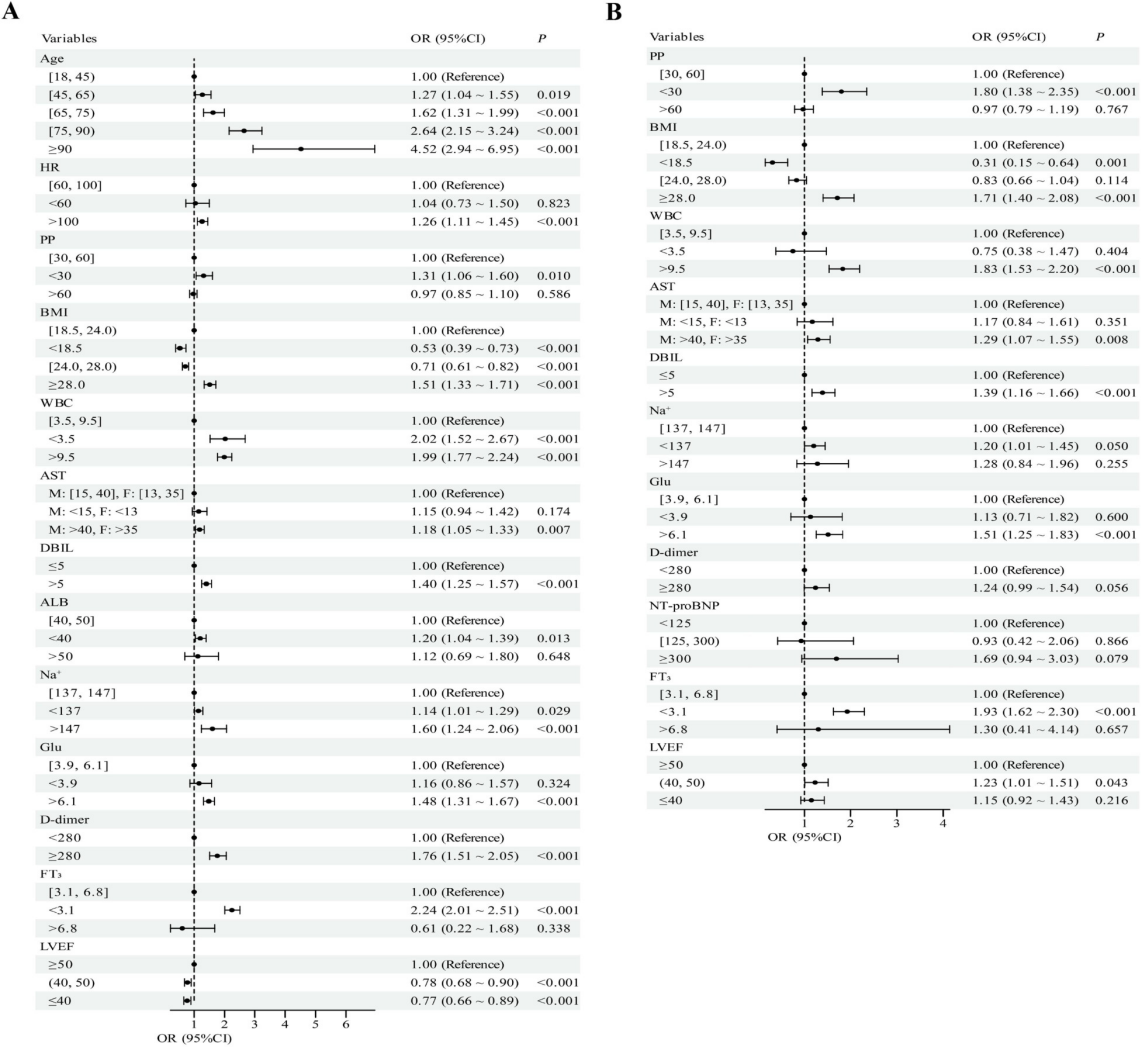
Multivariate logistic regression forest plots for all-cause mortality **(A)** and cardiac death **(B)** M: male, F: female.

Admission PP and 16 covariates were first screened in univariable logistic models for all-cause mortaliy and cardiac death. PP and LVEF were associated with both endpoints (*P* < 0.05; Supplementary Tables S3, S4). In multivariable models, PP <30 mmHg remained independently associated with higher risk of all-cause mortality (OR 1.31, 95% CI 1.06–1.60) and cardiac death (OR 1.80, 95% CI 1.38–2.35) relative to PP 30–60 mmHg. LVEF was also an independent predictor, whereas overweight (BMI 24.0 to <28.0 kg/m²) conferred protection and obesity (BMI ≥ 28 kg/m²) increased hazard. Additional independent correlates included advancing age, heart rate >100 bpm, albumin <40 g/L, serum sodium dysregulation, elevated D-dimer, and abnormal values of WBC, AST, DBIL, glucose and FT_3_ (Figure 2).

### Dose–response relationship between pulse pressure and mortality

**Figure 3:**
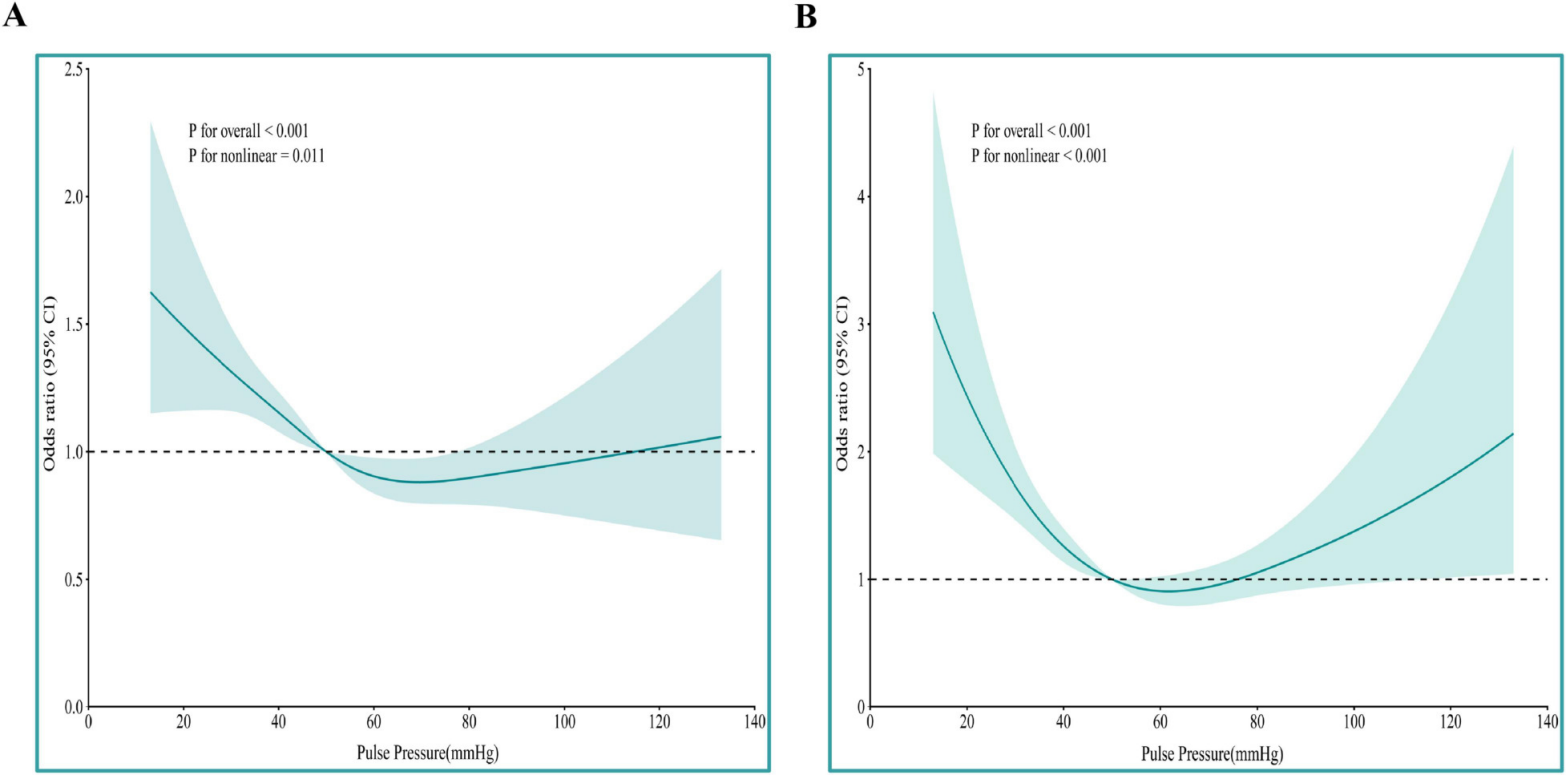
Nonlinear association of pulse pressure with all-cause mortality **(A)** and cardiac death **(B)** using restricted cubic splines. All-cause mortality adjusted for age, heart rate, BMI, WBC, AST, DBIL, ALB, Na^+^, Glu, D-dimer, FT_3_, LVEF in logistic regression model; Cardiac death adjusted for BMI, WBC, AST, DBIL, Glu, FT_3_, LVEF in logistic regression model. Reference value: 50 mmHg.

Restricted cubic splines (four knots, reference at median PP) adjusted for covariates retained in the multivariable model revealed significant non-linear relationships between PP and both all-cause mortality (*P*_non−linea*r*_ = 0.011) and cardiac death (*P*_non−linear_ < 0.001). Risk rose steeply as PP fell below 50 mmHg, plateaued thereafter for all-cause mortality (inverse J-shape), and trended upward again for cardiac death when PP exceeded approximately 80 mmHg (U-shape), although the latter did not reach statistical significance (Figure 3).

### Pulse pressure and in-hospital mortality cross LVEF phenotypes

#### Phenotype-specific associations

**Figure 4:**
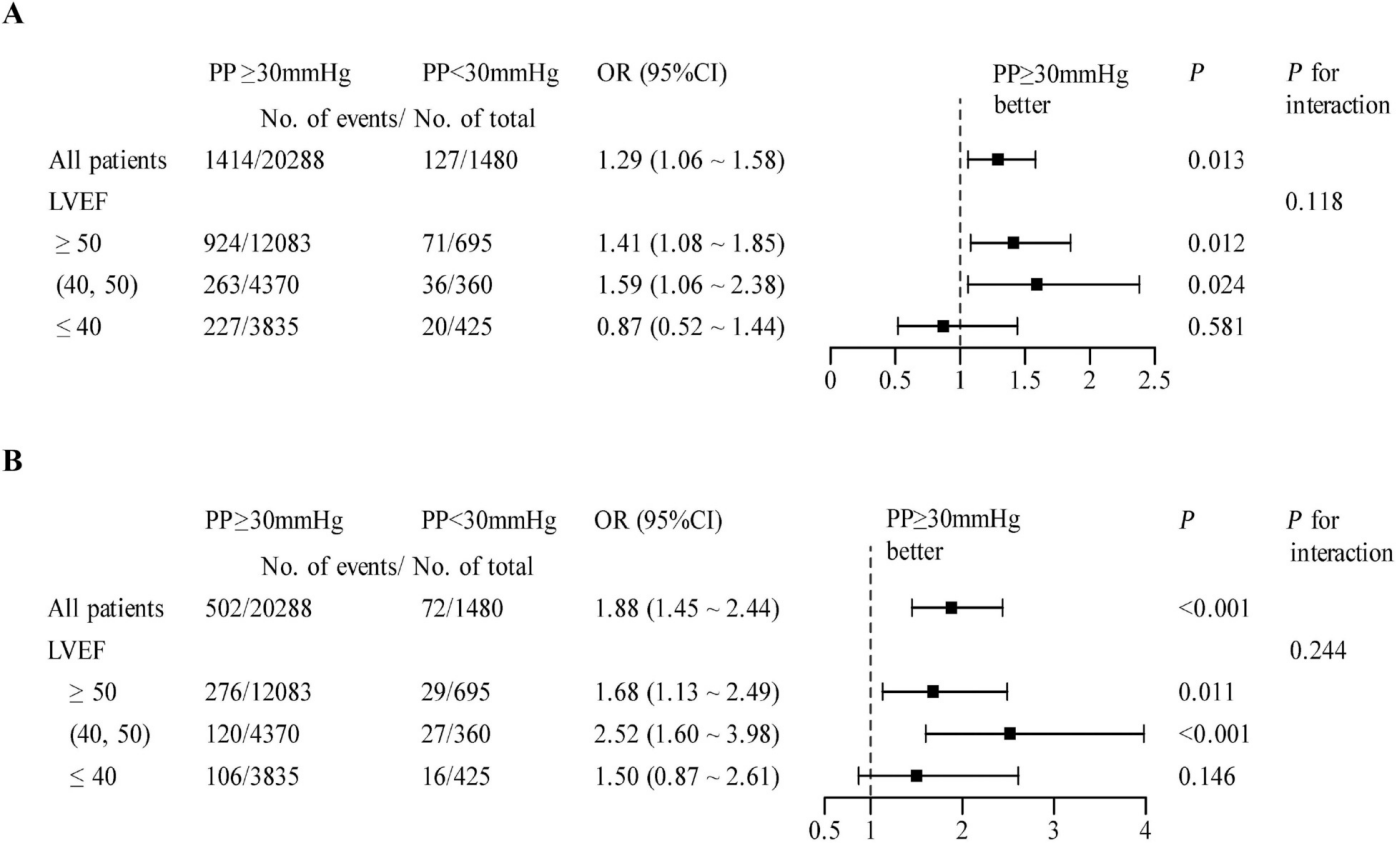
Association of pulse pressure with all-cause mortality **(A)** and cardiac death **(B)**, stratified by left ventricular ejection fraction.

The association between pulse pressure and in-hospital mortality was not modified by EF category (*P*_interaction_ > 0.05). In a prespecified subgroup analysis dichotomised at 30 mmHg, PP <30 mmHg conferred excess risk of both all-cause mortality and cardiac death among patients with LVEF ≥50% and 40%–49%; In HFrEF, the same threshold was linked to a numerically higher rate of cardiac death (OR 1.50, 95% CI 0.87–2.61), although this estimate did not achieve conventional statistical significance (Figure 4).

#### Dose–response splines by EF category

**Figure 5:**
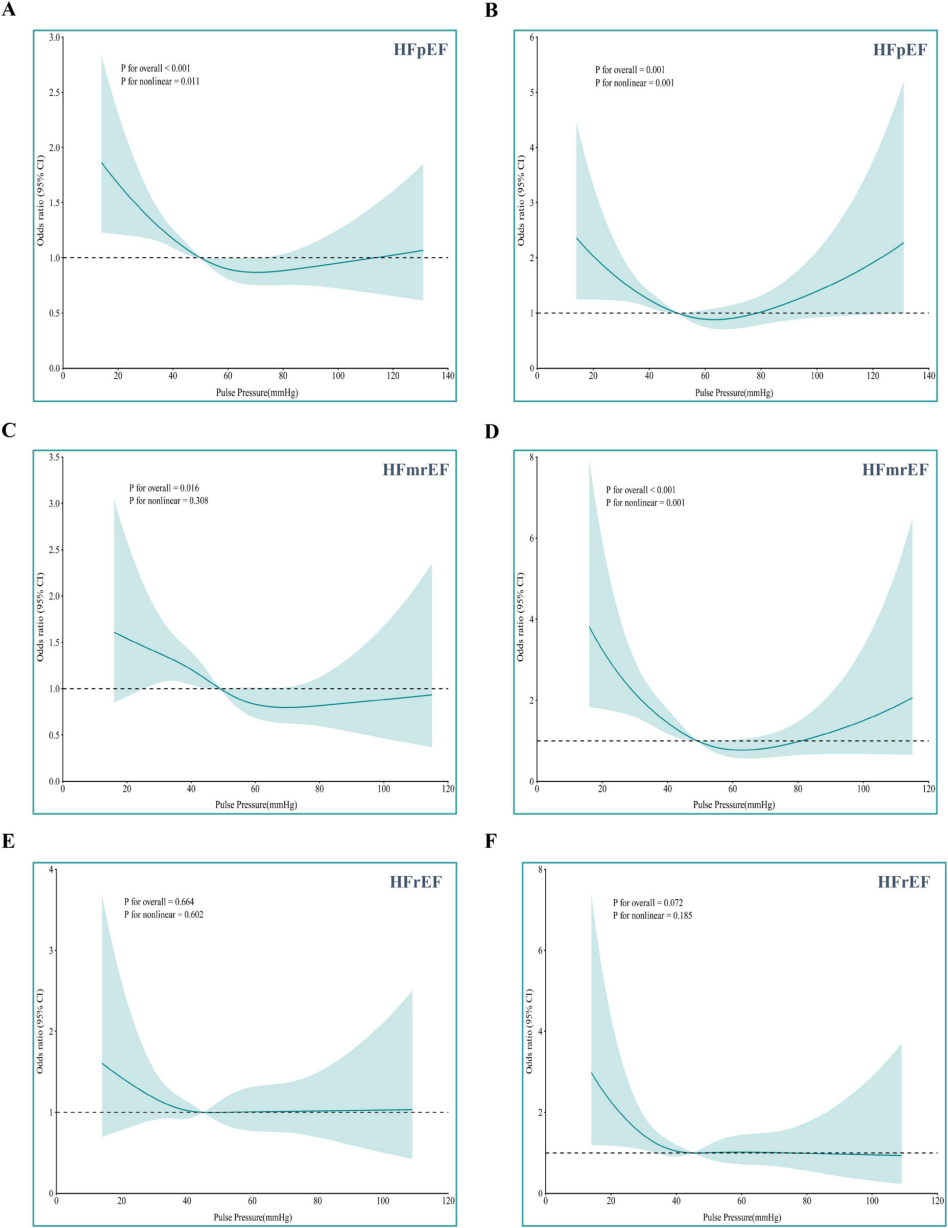
Nonlinear association of pulse pressure with all-cause mortality **(A,C,E)** and cardiac death **(B,D,F)** using restricted cubic splines in logistic regression model by left ventricular ejection fraction phenotype. **(A)**, adjusted for age, BMI, WBC, AST, DBIL, Na^+^, Glu, D-dimer, FT_3_; **(B)**, adjusted for BMI, WBC, ALT, DBIL, Glu, FT_3_; Reference value: 50 mmHg. **(C)**, adjusted for age, HR, BMI, WBC, DBIL, Glu, D-dimer, FT_3_; **(D)**, adjusted for HR, BMI, WBC, AST, Glu, FT_3_; Reference value: 49 mmHg. **(E)**, adjusted for age, HR, BMI, WBC, ALB, Na^+^, Glu, D-dimer, FT_3_; **(F)**, adjusted for WBC, ALB, Na^+^, Glu, FT_3_; Reference value: 45 mmHg.

Multivariate logistic regression analyses were performed separately for each EF-stratified subgroup (Supplementary Table S5, S6). Covariate-adjusted restricted cubic splines (4 knots) were constructed to characterize the relationship between pulse pressure and in-hospital mortality across heart failure subtypes (Figure 5). In HFpEF, PP exhibited a significant non-linear association with both all-cause mortality *(P*_non−linea*r*_ = 0.011) and cardiac death (*P*_non−linea*r*_ = 0.001): risk rose steeply below 50 mmHg and trended upward again at ≈80 mmHg. Among HFmrEF patients, the curve for all-cause mortality was monotonic but did not achieve non-linear significance (*P*_non−linea*r*_ = 0.308), whereas the pattern for cardiac death mirrored that observed in HFpEF. In HFrEF, although no significant associations were observed between PP and in-hospital all-cause mortality (*P*_overall_ = 0.664) or cardiac death (*P*_overall_ = 0.072), the shape of the curve indicated a relatively higher mortality risk in the range of low PP.

## Discussion

Heart failure represents the advanced and terminal stage of most cardiac disorders and constitutes one of the heaviest epidemiologic and economic burdens on health systems worldwide. Driven by the rising prevalence of coronary artery disease, hypertension, and diabetes-together with rapid population ageing in China-the HF epidemic continues to expand (2). Surveys indicate that in Asian countries, in-hospital mortality among HF patients ranges from 4.1% to 6.6% (1), with cardiovascular deaths accounting for the largest proportion, representing 71.5% of all-cause mortality in China (2).In the present hospital-based cohort, the observed in-hospital all-cause mortality was 7.08%, modestly higher than previously reported, with cardiac death comprising 37.29%. Several explanations underlie these findings. Firstly, the 10-year recruitment span captured an era during which earlier patient cohorts exhibited poor adherence to guidelines, with limited access to contemporary prognostic-improving agents such as angiotensin receptor-neprilysin inhibitors and sodium-glucose cotransporter 2 inhibitors, resulting in sub-optimal evidence-based care. Secondly, patients were enrolled from all hospital departments; variability in non-cardiologists' familiarity with HF diagnosis, trigger identification, volume status assessment, and pharmacologic protocols may have led to both misclassification and delayed treatment. Finally, vascular deaths (aortic dissection/rupture, stroke, pulmonary embolism) were not adjudicated as cardiac death. Thus, the current study aligns with the complexities of real-world clinical practice, enhancing the external validity of its findings.

Although PP has been repeatedly linked to adverse mid- and long-term outcomes in heart failure (12, 13), PP is conspicuously absent from widely used short-term risk models such as ADHERE, GWTG-HF, and PROTECT (14–16). Whether admission PP carries prognostic weight for fatal events during a heart-failure hospitalisation remains insufficiently interrogated. Initial explorations in this field have been derived from data of the HEARTSD registry, which indicated that the prognostic value of PP varies by HF phenotype (17): in patients with HFrEF, a PP below 50 mmHg was associated with a higher in-hospital mortality rate, yet PP itself was not an independent predictor of in-hospital mortality; in contrast, no significant correlation was observed between PP and short-term mortality risk in patients with HFpEF. The present study also focuses on the impact of admission PP on in-hospital mortality in HF patients, and its findings contradict those of previous reports. Specifically, low PP reamains robustly and independently associated with both all-cause mortality and cardiac death after rigorous adjustment for age, heart rate, serum sodium, and other established determinants of early post-admission outcome. The discrepancies between the above findings may stem from differences in the inclusion criteria for study subjects and inconsistencies in the cut-off values used to define low PP. Our cohort exhibited a clinically intriguing baseline pattern: the low PP group had the youngest mean age and the lowest prevalence of traditional atherosclerotic comorbidities, but the lowest LVEF, while the high PP group showed the opposite pattern. This finding is highly consistent with two prior studies by Teng TK et al. (1) and Lu H et al. (2), both of which reported significantly lower rates of hypertension, diabetes, coronary atherosclerotic heart disease, and stroke in patients with reduced PP compared with those with elevated PP. Furthermore, a recent nationwide cohort study from the Chinese Cardiovascular Association Heart Failure Center Registry (3) also confirmed that patients with HFrEF had lower incidence of the aforementioned four comorbidities and lower blood pressure levels than those with HFpEF.

Previous investigators have confirmed that reduced PP elevates mortality risk in patients with acute decompensated HF or advanced HF (12, 18). Additionally, PP has been identified as a robust and independent predictor of survival following extracorporeal cardiopulmonary resuscitation (eCPR), aiding clinical decision-making in subsequent management. As a practical indirect indicator of hemodynamic stability, low PP after eCPR typically portends a higher risk of death (19). In anesthesiology, reduced PP similarly forecasts major adverse events, underscoring its utility as an instantaneous surrogate of cardiac output reserve (20). From a pathophysiological perspective, pulse pressure is primarily determined by two factors: arterial compliance and left ventricular stroke volume. High PP in elderly patients is predominantly driven by age-related arterial stiffening and increased peripheral resistance, which is closely associated with atherosclerotic comorbidities and typically occurs in the setting of preserved or mildly reduced LVEF. In contrast, low PP in young HF patients is a direct hemodynamic marker of severely impaired systolic function and diminished stroke volume, rather than vascular pathology. Mechanistically, reduced PP reflects diminished stroke volume and impaired contractile reserve, triggering sympathetic and neuro-humoral activation, ischaemia, and maladaptive remodelling, culminating in severe impairment of cardiac systolic and diastolic function and death (21). Conversely, a wider PP in this context denotes stable hemodynamics and preserved stroke volume, and relatively stable clinical condition in patients. This also reinforces our primary conclusion that low pulse pressure at admission serves as a powerful independent prognostic marker, reflecting intrinsic myocardial dysfunction—even in patients without traditional cardiovascular risk factors—and similarly explains the non-significant association observed for higher PP [all-cause mortality OR 0.97 [95% CI 0.85–1.10]; cardiac death OR 0.97 [95% CI 0.79–1.19]]. We observed no statistically significant interaction between pulse pressure and ejection fraction phenotype with respect to in-hospital mortality (*P*_interaction_ >0.05). Across HFrEF, HFmrEF and HFpEF, PP <30 mmHg consistently identified patients at heightened risk of fatal events. Synthesizing previous research, the lowest mortality risk is consistently observed when PP is approximately 50 mmHg (8, 9, 22), a finding corroborated by the RCS curve in the present study. In HFpEF, both excessively low and widened PP showed a trend toward increased risk of cardiac mortality, which echoes previous observations (7, 8, 22).

Notably, in our cohort, 58.70% of patients had HFpEF, with the highest in-hospital mortality of 7.79%; 21.73% had HFmrEF, with an in-hospital mortality of 6.32%; and 19.57% had HFrEF, with an in-hospital mortality of 5.8%. This contrasts with previous conclusions that HFrEF constitutes the largest proportion of HF cases with the highest mortality (2, 23). This discrepancy is probably multifactorial. Firstly, prior studies have primarily focused on patients treated in cardiology departments, whereas HFpEF patients, who frequently have comorbidities such as anemia, stroke, and chronic obstructive pulmonary disease, are more likely to be admitted to non-cardiology departments, leading to delayed diagnosis and management. In addition, evidence-based pharmacotherapies for HFpEF are limited and underutilized, and this population tends to be older with a rising burden of non-cardiovascular mortality (2, 24). Simultaneously, the measurement of EF in the present study was not standardized; values obtained via the Teichholz method may be inaccurate in patients with coronary artery disease, hypertrophic cardiomyopathy, or left ventricular aneurysms, potentially overestimating EF and leading to misclassification of HF subtypes, with an over representation of HFpEF. Future prospective studies should employ standardized, protocol-defined methods to validate our observations.

The inverse association between overweight and in-hospital mortality—observed in the overall cohort and preserved across all LVEF strata—reinforces the “obesity paradox” repeatedly documented in HF (2, 25, 26). Plausible mechanistic explanations include: (i) selection bias, whereby obese individuals present earlier with prodromal symptoms, undergo timelier diagnostic work-up, and receive more intensive concomitant treatment for hypertension, diabetes, and dyslipidaemia; (ii) Lean patients tend to have insufficient metabolic reserves. Severe heart failure itself is a catabolic state, and coupled with poor dietary intake, most suffer from malnutrition, resulting in inadequate energy to meet demands (25, 26). In this study, patients with BMI <18.5 kg/m² accounted for only 4.5%, which was insufficient to fully demonstrate that low body weight or cachexia increases the risk of death in heart failure (27). Further analysis in prospective cohorts is warranted.

## Strengths and limitations

Although the present study is an observational analysis, it possesses notable strengths. Our design offset by consecutive real-world capture of incident, readmitted, and in-hospital-diagnosed heart-failure cases enhancing generalizability. Our data confirmed admission PP independently predicted in-hospital mortality in HF patients without phenotype heterogeneity. Of particular importance, this study provides novel and valuable evidence for the field of HFmrEF, where prior evidence has been scarce. Nevertheless, several limitations merit consideration. First, the retrospective design precludes causal inference and remains vulnerable to unmeasured confounding. Although in-hospital patients included multiple ethnicities, data were collected from a single tertiary hospital, failing to cover a broader population of primary care patients, thus limiting external validity. Verification through larger-scale multicenter prospective randomized controlled trials is needed. Second, covariate adjustment was limited to variables selected by the random-forest algorithm; pharmacologic therapies and systolic blood pressure—both of which may act as confounders or effect modifiers of the PP-mortality relation (8, 21, 28, 29)—were not included, leaving residual distortion possible. This might explain why reduced EF emerged as a “protective” factor for all-cause mortality and why PP lost significance in HFrEF. These factors should be considered in subsequent studies, with subgroup analyses to explore effects when necessary. Third, the analysis was restricted to the first pulse-pressure value recorded after admission; because this measure relied on routine clinical documentation, we cannot guarantee that all blood-pressure readings were obtained under standardized, optimal conditions, introducing potential misclassification bias. For subsequent studies, predefining standardized blood pressure measurement protocols and investigating PP variability within the first 24 h after admission as well as the mean in-hospital PP—both of which are associated with short-term disease prognosis (11, 19, 30)—may enable a more precise delineation of the prognostic value of PP in heart failure. Fourth, LVEF measurements were not synchronized with blood pressure readings used to calculate pulse pressure. As a retrospective cohort study, we were unable to align the timing of these two measurements to the exact hour. Although the majority of measurements were performed within 24 h of admission, some patients could not undergo transthoracic echocardiography within this timeframe due to clinical contraindications. This discrepancy may have introduced measurement bias and potentially attenuated the association between pulse pressure and LVEF.

## Conclusion

Admission pulse pressure is an independent determinant of in-hospital all-cause mortality and cardiac death among patients with heart failure. A low pulse pressure robustly identifies individuals at heightened risk, and this association is not modified by left-ventricular ejection fraction. Designating pulse pressure as a key clinical monitoring indicator, strengthening training on standardized in-hospital diagnosis and treatment procedures for heart failure, early identification of signs of disease deterioration, implementation of effective interventions, and necessary multidisciplinary collaborative management may open a new pathway to reduce in-hospital mortality and improve prognosis in patients with heart failure.

## Data Availability

The raw data supporting the conclusions of this article will be made available by the authors, without undue reservation.

## References

[1] Savarese G Becher PM Lund LH Seferovic P Rosano GMC Coats AJS. Global burden of heart failure: a comprehensive and updated review of epidemiology. Cardiovasc Res. (2023) 118(17):3272–87. 10.1093/cvr/cvac013 35150240

[2] Wang H Li Y Chai K Long Z Yang Z Du M Mortality in patients admitted to hospital with heart failure in China: a nationwide cardiovascular association database-heart failure centre registry cohort study. Lancet Glob Health. (2024) 12(4):e611–e22. 10.1016/s2214-109x(23)00605-8 38485428

[3] Fang LX Wu YH Yao T Wang ZN Qian S Jiang T Use of pulse pressure Index for cardiovascular outcomes assessment and development of a coronary heart disease model for the elderly. BMC Cardiovasc Disord. (2025) 25(1):297. 10.1186/s12872-025-04641-8 40251528 PMC12007300

[4] Sharashova E Gerdts E Ball J Schnabel RB Stylidis M Tiwari S Long-Term pulse pressure trajectories and risk of incident atrial fibrillation: the tromsø study. Eur Heart J. (2025) 46(14):1291–300. 10.1093/eurheartj/ehaf005 39820670 PMC11973555

[5] Zhang Z Gu X Tang Z Guan S Liu H Wu X Associations of blood pressure components with risks of cardiovascular events and all-cause death in a Chinese population: a prospective study. J Clin Hypertens (Greenwich). (2022) 24(7):825–37. 10.1111/jch.14529 35748650 PMC9278591

[6] Jackson CE Castagno D Maggioni AP Køber L Squire IB Swedberg K Differing prognostic value of pulse pressure in patients with heart failure with reduced or preserved ejection fraction: results from the maggic individual patient meta-analysis. Eur Heart J. (2015) 36(18):1106–14. 10.1093/eurheartj/ehu490 25616644

[7] Teng TK Tay WT Dahlstrom U Benson L Lam CSP Lund LH. Different relationships between pulse pressure and mortality in heart failure with reduced, mid-range and preserved ejection fraction. Int J Cardiol. (2018) 254:203–9. 10.1016/j.ijcard.2017.09.187 29407092

[8] Laskey WK Wu J Schulte PJ Hernandez AF Yancy CW Heidenreich PA Association of arterial pulse pressure with long-term clinical outcomes in patients with heart failure. JACC Heart Fail. (2016) 4(1):42–9. 10.1016/j.jchf.2015.09.012 26656142

[9] Lu H Kondo T Claggett BL Vaduganathan M Neuen BL Beldhuis IE Systolic blood pressure and pulse pressure in heart failure: pooled participant-level analysis of 4 trials. J Am Coll Cardiol. (2025) 85(7):710–22. 10.1016/j.jacc.2024.11.007 39745404

[10] Wang L Liu Y Zhang S Li J Cui Y Yun Y Admission pulse pressure and in-hospital mortality in type a acute aortic dissection: result from a Chinese study in stable patients on admission. Eur J Med Res. (2025) 30(1):203. 10.1186/s40001-025-02475-w 40134032 PMC11938771

[11] Han J Santo D Mathew P Hibbert R Grinstein B Belkin J Pulse pressure response to inotrope therapy in cardiogenic shock: a subanalysis of the doremi trial. JACC Heart Fail. (2024) 12(6):1126–7. 10.1016/j.jchf.2024.03.013 38839155

[12] Chen C Chen X Chen S Wu Y He X Zhao J Prognostic implication of admission mean and pulse pressure in acute decompensated heart failure with different phenotypes. Am J Hypertens. (2023) 36(4):217–25. 10.1093/ajh/hpac130 36520093

[13] Hu H Liu Z Zeng J Jiang M. Impact of initial heart rate, diastolic pressure, and pulse pressure on prognostic outcomes in heart failure patients with mildly reduced ejection fraction. Int J Gen Med. (2025) 18:403–14. 10.2147/ijgm.S487722 39881953 PMC11776420

[14] Fonarow GC Adams KF Jr. Abraham WT Yancy CW Boscardin WJ. Risk stratification for in-hospital mortality in acutely decompensated heart failure: classification and regression tree analysis. Jama. (2005) 293(5):572–80. 10.1001/jama.293.5.572 15687312

[15] O'Connor CM Mentz RJ Cotter G Metra M Cleland JG Davison BA The protect in-hospital risk model: 7-day outcome in patients hospitalized with acute heart failure and renal dysfunction. Eur J Heart Fail. (2012) 14(6):605–12. 10.1093/eurjhf/hfs029 22535795

[16] Peterson PN Rumsfeld JS Liang L Albert NM Hernandez AF Peterson ED A validated risk score for in-hospital mortality in patients with heart failure from the American Heart Association get with the guidelines program. Circ Cardiovasc Qual Outcomes. (2010) 3(1):25–32. 10.1161/circoutcomes.109.854877 20123668

[17] Aljohar A Alhabib K AlFaleh H Hersi A Habeeb WA Ullah A The prognostic impact of pulse pressure in acute heart failure: insights from the hearts registry. J Saudi Heart Assoc. (2020) 32(2):263–73. 10.37616/2212-5043.1025 33154927 PMC7640568

[18] Dobre D Kjekshus J Rossignol P Girerd N Benetos A Dickstein K Heart rate, pulse pressure and mortality in patients with myocardial infarction complicated by heart failure. Int J Cardiol. (2018) 271:181–5. 10.1016/j.ijcard.2018.05.017 30223350

[19] Rilinger J Riefler AM Bemtgen X Jäckel M Zotzmann V Biever PM Impact of pulse pressure on clinical outcome in extracorporeal cardiopulmonary resuscitation (ecpr) patients. Clin Res Cardiol. (2021) 110(9):1473–83. 10.1007/s00392-021-01838-7 33779810 PMC8405467

[20] Ackland GL Abbott TEF Pearse RM Karmali SN Whittle J Minto G. Arterial pulse pressure and postoperative morbidity in high-risk surgical patients. Br J Anaesth. (2018) 120(1):94–100. 10.1016/j.bja.2017.11.009 29397143

[21] Naka KK Ikonomidis I. Brachial pulse pressure in heart failure: simple to measure but Complex to interpret. Eur Heart J. (2019) 40(26):e8–e10. 10.1093/eurheartj/ehv005 25694463

[22] Tokitsu T Yamamoto E Hirata Y Kusaka H Fujisue K Sueta D Clinical significance of pulse pressure in patients with heart failure with preserved left ventricular ejection fraction. Eur J Heart Fail. (2016) 18(11):1353–61. 10.1002/ejhf.559 27197000

[23] Zhang Y Zhang J Butler J Yang X Xie P Guo D Contemporary epidemiology, management, and outcomes of patients hospitalized for heart failure in China: results from the China heart failure (China-hf) registry. J Card Fail. (2017) 23(12):868–75. 10.1016/j.cardfail.2017.09.014 29029965

[24] McDonagh TA Metra M Adamo M Gardner RS Baumbach A Böhm M 2023 Focused update of the 2021 esc guidelines for the diagnosis and treatment of acute and chronic heart failure. Eur Heart J. (2023) 44(37):3627–39. 10.1093/eurheartj/ehad195 37622666

[25] Mahajan R Stokes M Elliott A Munawar DA Khokhar KB Thiyagarajah A Complex interaction of obesity, intentional weight loss and heart failure: a systematic review and meta-analysis. Heart. (2020) 106(1):58–68. 10.1136/heartjnl-2019-314770 31530572

[26] Powell-Wiley TM Poirier P Burke LE Després JP Gordon-Larsen P Lavie CJ Obesity and cardiovascular disease: a scientific statement from the American Heart Association. Circulation. (2021) 143(21):e984–e1010. 10.1161/cir.0000000000000973 33882682 PMC8493650

[27] Kenchaiah S Pocock SJ Wang D Finn PV Zornoff LA Skali H Body mass Index and prognosis in patients with chronic heart failure: insights from the candesartan in heart failure: assessment of reduction in mortality and morbidity (charm). Program. Circulation. (2007) 116(6):627–36. 10.1161/circulationaha.106.679779 17638930

[28] Suzuki K Claggett B Minamisawa M Nochioka K Mitchell GF Anand IS Pulse pressure, prognosis, and influence of sacubitril/valsartan in heart failure with preserved ejection fraction. Hypertension. (2021) 77(2):546–56. 10.1161/hypertensionaha.120.16277 33356401

[29] Tang KS Medeiros ED Shah AD. Wide pulse pressure: a clinical review. J Clin Hypertens (Greenwich). (2020) 22(11):1960–7. 10.1111/jch.14051 32986936 PMC8029839

[30] Kamieniarz-Mędrygał M Łukomski T Kaźmierski R. Short-Term outcome after ischemic stroke and 24-H blood pressure variability: association and predictors. Hypertens Res. (2021) 44(2):188–96. 10.1038/s41440-020-00534-9 32801313

