# Supplementary material for: Low admission pulse pressure and increased in-hospital mortality in patients with heart failure
Source: Front Cardiovasc Med. 2026 Apr 30;13:1747168. doi: 10.3389/fcvm.2026.1747168 (PMC13171785; doi:10.3389/fcvm.2026.1747168)
Supplement: Supplementary file 1 [file Datasheet1.docx]

Supplementary Material

##
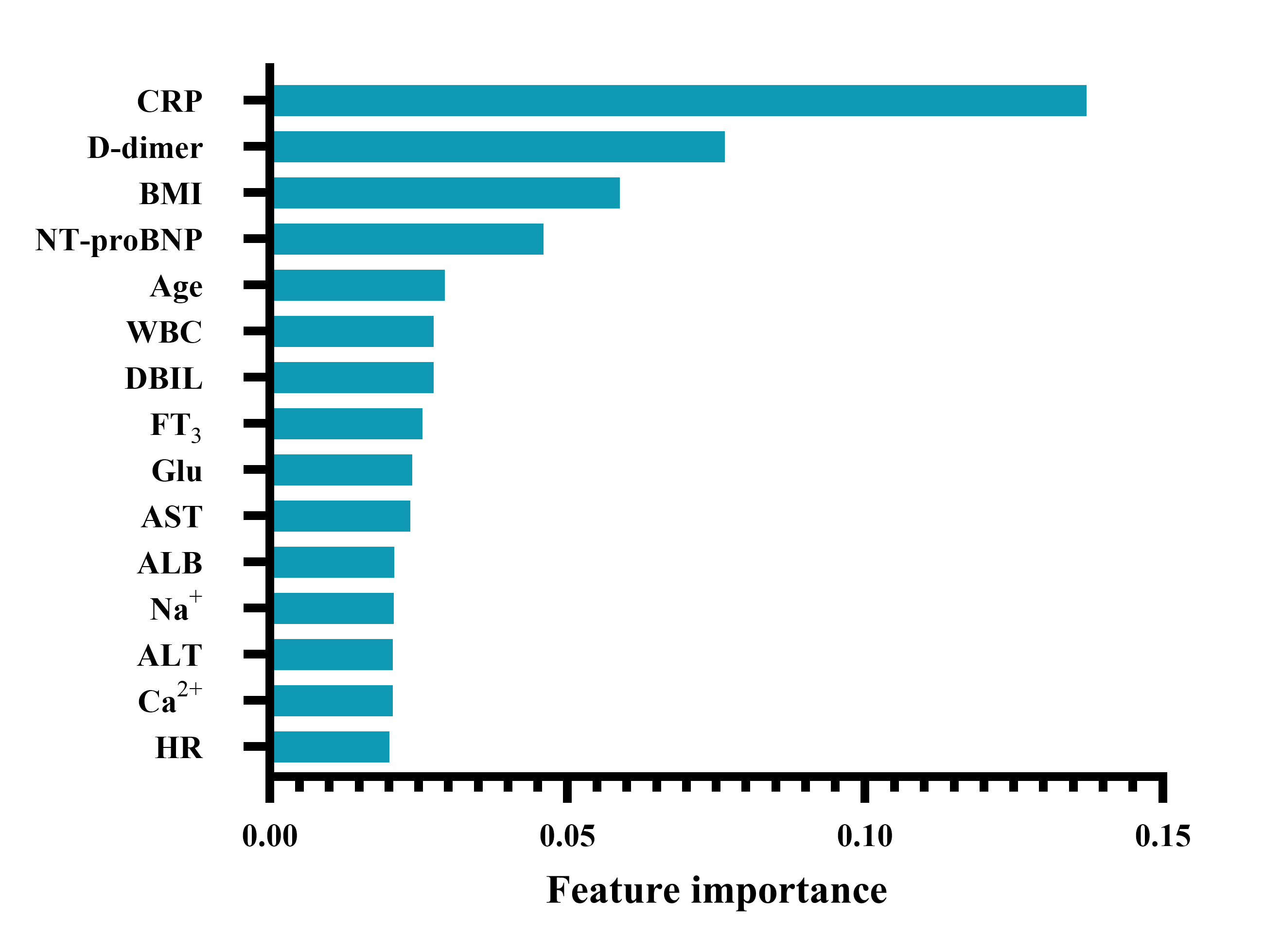
Supplementary Figure

# Supplementary Tables

**Supplementary Table 1.** Clinical indicators collected in the study.

| Number | Clinical indicators |  | Number | Clinical indicators |
| --- | --- | --- | --- | --- |
| 1 | All-cause mortality |  | 28 | Creatinine, Cr, μmol/L |
| 2 | Cardiac death |  | 29 | Urea, Ur, μmol/L |
| Demographics | |  | 30 | Cystatin C, Cys C, mg/L |
| 3 | Sex |  | 31 | estimated Glomerular filtration rate, eGFR, mL/min/1.73 m^2^ |
| 4 | Age, years |  | 32 | Total cholesterol, TC, mmol/L |
| Medical history | |  | 33 | Triglyceride, TG, mmol/L |
| 5 | Smoking |  | 34 | High density lipoprotein-cholesterol, HDL-C, mmol/L |
| 6 | Drinking |  | 35 | Low density lipoprotein-cholesterol, LDL-C, mmol/L |
| 7 | Hypertension, HTN |  | 36 | C-reactive protein, CRP, mg/L |
| 8 | Diabetes mellitus, DM |  | 37 | Prothrombin time, PT, second |
| 9 | Coronary atherosclerotic heart disease, CAD |  | 38 | Activated partial thromboplastin time, APTT, second |
| 10 | Cerebral Infarction, CI |  | 39 | Thrombin time, TT, second |
| Clinical manifestation and vitals | |  | 40 | International Normalized ratio, INR |
| 11 | Lower extremity edema |  | 41 | D-dimer, D-D, ng/mL |
| 12 | Heart rate, HR, times/minute |  | 42 | N‑terminal pro‑BNP, NT‑proBNP, ng/L |
| 13 | Respiratory rate, R, times/minute |  | 43 | Thyroid stimulating hormone, TSH, mIU/L |
| 14 | Pulse Pressure, PP, mmHg |  | 44 | Free thyroxine, FT_4_, pmol/L |
| 15 | Body mass index, BMI, kg/m^2^ |  | 45 | Free triiodothyronine, FT_3_, pmol/L |
| Laboratory tests | |  | Echocardiogram result | |
| 16 | White blood cell, WBC, 10^9^/L |  | 46 | Left ventricular ejection fraction, LVEF, % |
| 17 | Hemoglobin, Hb, g/L |  | Pre-hospital and in-hospital medication | |
| 18 | Serum potassium, K^+^, mmol/L |  | 47 | Loop diuretics |
| 19 | Serum sodium, Na^+^, mmol/L |  | 48 | Digoxin; Cedilanid |
| 20 | Serum calcium, Ca^2+^, mmol/L |  | 49 | Calcium-channel blockers(Dihydropyridines), CCB(DHPs) |
| 21 | Glucose, Glu, mmol/L |  | 50 | Sodium‑glucose co‑transporter 2 inhibitors, SGLT2i |
| 22 | Alanine amiNotransferase, ALT, U/L |  | 51 | Beta-blockers |
| 23 | Aspartate amiNotransferase, AST, U/L |  | 52 | Spironolactone |
| 24 | Direct bilirubin, DBIL, μmol/L |  | 53 | Angiotensin-converting enzyme inhibitors, ACEI; Angiotensin II receptor antagonists, ARB;  Angiotensin receptor neprilysin inhibitor, ARNI |
| 25 | Indirect Bilirubin, IBIL, μmol/L |  |  |  |
| 26 | Albumin, ALB, g/L |  |  |  |
| 27 | Uric acid, UA, μmol/L |  |  |  |

eGFR was calculated based on the 2021 Chronic Kidney Disease Epidemiology Collaboration creatinine-cystatin C equations.

**Supplementary Table 2.** Dummy variable assignment table.

| Variables | Assignment rules |
| --- | --- |
| All-cause mortality | No=0, Yes=1 |
| Cardiac death | No=0, Yes=1 |
| Age | [18, 45)=0, [45, 65)=1, [65, 75)=2, [75, 90)=3, ≥90=4 |
| Heart Rate | [60, 100]=0, <60=1, >100=2 |
| Pulse Pressure | [30, 60]=0, <30=1, >60=2 |
| BMI | [18.5, 24.0)=0, <18.5=1, [24.0, 28.0)=2, ≥28.0=3 |
| WBC | [3.5, 9.5]=0, <3.5=1, >9.5=2 |
| ALT | Male: [9, 50]=0, <9=1, >50=2; Femal: [7, 40]=0, <7=1, >40=2 |
| AST | Male: [15, 40]=0, <15=1, >40=2; Female: [13, 35]=0, <13=1, >35=2 |
| DBIL | ≤5=0, >5=1 |
| ALB | [40, 55]=0, <40=1, >55=2 |
| Na^+^ | [137, 147]=0, <137=1, >147=2 |
| Ca^2+^ | [2.11, 2.52]=0, <2.11=1, >2.52=2 |
| Glu | 0=[3.9, 6.1], 1=<3.9, 2=>6.1 |
| CRP | ≤10=0, >10=1 |
| D-dimer | <280=0, ≥280=1 |
| NT‑proBNP | <125=0, [125, 300)=1, ≥300=2 |
| FT_3_ | [3.1, 6.8]=0, <3.1=1, >6.8=2 |
| LVEF | ≥50=0, (40, 50)=1, ≤40=2 |

**Supplementary Table 3.** Results of univariate and multivariate logistic regression analyses for all-cause mortality.

| Variables | univariate | | | | |  | multivariate | | | | |
| --- | --- | --- | --- | --- | --- | --- | --- | --- | --- | --- | --- |
|  | β | S.E | Z | *P* | OR (95%CI) |  | β | S.E | Z | *P* | OR (95%CI) |
| Age |  |  |  |  |  |  |  |  |  |  |  |
| [18, 45) |  |  |  |  | 1.00 (Reference) |  |  |  |  |  | 1.00 (Reference) |
| [45, 65) | 0.09 | 0.10 | 0.94 | 0.345 | 1.10 (0.90 ~ 1.33) |  | 0.24 | 0.10 | 2.34 | 0.019 | 1.27 (1.04 ~ 1.55) |
| [65, 75) | 0.37 | 0.10 | 3.67 | <0.001 | 1.45 (1.19 ~ 1.78) |  | 0.48 | 0.11 | 4.50 | <0.001 | 1.62 (1.31 ~ 1.99) |
| [75, 90) | 0.95 | 0.10 | 9.63 | <0.001 | 2.58 (2.13 ~ 3.13) |  | 0.97 | 0.10 | 9.30 | <0.001 | 2.64 (2.15 ~ 3.24) |
| ≥90 | 1.65 | 0.21 | 7.98 | <0.001 | 5.20 (3.47 ~ 7.80) |  | 1.51 | 0.22 | 6.88 | <0.001 | 4.52 (2.94 ~ 6.95) |
| HR |  |  |  |  |  |  |  |  |  |  |  |
| [60, 100] |  |  |  |  | 1.00 (Reference) |  |  |  |  |  | 1.00 (Reference) |
| <60 | 0.03 | 0.18 | 0.19 | 0.849 | 1.03 (0.73 ~ 1.46) |  | 0.04 | 0.18 | 0.22 | 0.823 | 1.04 (0.73 ~ 1.50) |
| >100 | 0.64 | 0.06 | 10.26 | <0.001 | 1.90 (1.68 ~ 2.14) |  | 0.23 | 0.07 | 3.42 | <0.001 | 1.26 (1.11 ~ 1.45) |
| PP |  |  |  |  |  |  |  |  |  |  |  |
| [30, 60] |  |  |  |  | 1.00 (Reference) |  |  |  |  |  | 1.00 (Reference) |
| <30 | 0.25 | 0.10 | 2.52 | 0.012 | 1.28 (1.06 ~ 1.55) |  | 0.27 | 0.10 | 2.56 | 0.010 | 1.31 (1.06 ~ 1.60) |
| >60 | 0.08 | 0.06 | 1.36 | 0.175 | 1.09 (0.96 ~ 1.23) |  | -0.04 | 0.07 | -0.54 | 0.586 | 0.97 (0.85 ~ 1.10) |
| BMI |  |  |  |  |  |  |  |  |  |  |  |
| [18.5, 24.0) |  |  |  |  | 1.00 (Reference) |  |  |  |  |  | 1.00 (Reference) |
| <18.5 | -0.51 | 0.16 | -3.25 | 0.001 | 0.60 (0.44 ~ 0.82) |  | -0.63 | 0.16 | -3.93 | <0.001 | 0.53 (0.39 ~ 0.73) |
| [24.0, 28.0) | -0.50 | 0.07 | -6.97 | <0.001 | 0.61 (0.53 ~ 0.70) |  | -0.35 | 0.07 | -4.70 | <0.001 | 0.71 (0.61 ~ 0.82) |
| ≥28.0 | 0.23 | 0.06 | 3.72 | <0.001 | 1.26 (1.11 ~ 1.42) |  | 0.41 | 0.06 | 6.33 | <0.001 | 1.51 (1.33 ~ 1.71) |
| WBC |  |  |  |  |  |  |  |  |  |  |  |
| [3.5, 9.5] |  |  |  |  | 1.00 (Reference) |  |  |  |  |  | 1.00 (Reference) |
| <3.5 | 0.87 | 0.14 | 6.35 | <0.001 | 2.40 (1.83 ~ 3.14) |  | 0.70 | 0.14 | 4.90 | <0.001 | 2.02 (1.52 ~ 2.67) |
| >9.5 | 1.01 | 0.06 | 18.24 | <0.001 | 2.73 (2.45 ~ 3.05) |  | 0.69 | 0.06 | 11.45 | <0.001 | 1.99 (1.77 ~ 2.24) |
| ALT |  |  |  |  |  |  |  |  |  |  |  |
| M: [9, 50], F: [7, 40] |  |  |  |  | 1.00 (Reference) |  |  |  |  |  |  |
| M: <9, F: <7 | 0.25 | 0.12 | 2.11 | 0.035 | 1.28 (1.02 ~ 1.61) |  |  |  |  |  |  |
| M: >50, F: >40 | 0.39 | 0.06 | 6.56 | <0.001 | 1.48 (1.32 ~ 1.66) |  |  |  |  |  |  |
| AST |  |  |  |  |  |  |  |  |  |  |  |
| M: [15, 40], F: [13, 35] |  |  |  |  | 1.00 (Reference) |  |  |  |  |  | 1.00 (Reference) |
| M: <15, F: <13 | 0.10 | 0.10 | 0.96 | 0.335 | 1.10 (0.90 ~ 1.35) |  | 0.14 | 0.11 | 1.36 | 0.174 | 1.15 (0.94 ~ 1.42) |
| M: >40, F: >35 | 0.61 | 0.06 | 11.03 | <0.001 | 1.84 (1.65 ~ 2.06) |  | 0.16 | 0.06 | 2.68 | 0.007 | 1.18 (1.05 ~ 1.33) |
| DBIL |  |  |  |  |  |  |  |  |  |  |  |
| ≤5 |  |  |  |  | 1.00 (Reference) |  |  |  |  |  | 1.00 (Reference) |
| >5 | 0.41 | 0.06 | 7.41 | <0.001 | 1.51 (1.35 ~ 1.68) |  | 0.34 | 0.06 | 5.71 | <0.001 | 1.40 (1.25 ~ 1.57) |
| ALB |  |  |  |  |  |  |  |  |  |  |  |
| [40, 55] |  |  |  |  | 1.00 (Reference) |  |  |  |  |  | 1.00 (Reference) |
| <40 | 0.69 | 0.07 | 9.88 | <0.001 | 1.99 (1.74 ~ 2.29) |  | 0.19 | 0.07 | 2.48 | 0.013 | 1.20 (1.04 ~ 1.39) |
| >55 | 0.47 | 0.23 | 2.01 | 0.045 | 1.60 (1.01 ~ 2.54) |  | 0.11 | 0.24 | 0.46 | 0.648 | 1.12 (0.69 ~ 1.80) |
| Na^+^ |  |  |  |  |  |  |  |  |  |  |  |
| [137, 147] |  |  |  |  | 1.00 (Reference) |  |  |  |  |  | 1.00 (Reference) |
| <137 | 0.54 | 0.06 | 9.64 | <0.001 | 1.72 (1.54 ~ 1.92) |  | 0.13 | 0.06 | 2.18 | 0.029 | 1.14 (1.01 ~ 1.29) |
| >147 | 0.85 | 0.12 | 6.96 | <0.001 | 2.33 (1.84 ~ 2.96) |  | 0.47 | 0.13 | 3.59 | <0.001 | 1.60 (1.24 ~ 2.06) |
| Ca^2+^ |  |  |  |  |  |  |  |  |  |  |  |
| [2.11, 2.52] |  |  |  |  | 1.00 (Reference) |  |  |  |  |  |  |
| <2.11 | 0.54 | 0.05 | 10.04 | <0.001 | 1.71 (1.54 ~ 1.90) |  |  |  |  |  |  |
| >2.52 | 0.24 | 0.18 | 1.38 | 0.167 | 1.28 (0.90 ~ 1.81) |  |  |  |  |  |  |
| Glu |  |  |  |  |  |  |  |  |  |  |  |
| [3.9, 6.1] |  |  |  |  | 1.00 (Reference) |  |  |  |  |  | 1.00 (Reference) |
| <3.9 | 0.17 | 0.15 | 1.14 | 0.255 | 1.19 (0.88 ~ 1.59) |  | 0.15 | 0.15 | 0.99 | 0.324 | 1.16 (0.86 ~ 1.57) |
| >6.1 | 0.65 | 0.06 | 11.29 | <0.001 | 1.92 (1.72 ~ 2.15) |  | 0.39 | 0.06 | 6.35 | <0.001 | 1.48 (1.31 ~ 1.67) |
| CRP |  |  |  |  |  |  |  |  |  |  |  |
| ≤10 |  |  |  |  | 1.00 (Reference) |  |  |  |  |  |  |
| >10 | 0.40 | 0.08 | 5.06 | <0.001 | 1.49 (1.28 ~ 1.74) |  |  |  |  |  |  |
| D-dimer |  |  |  |  |  |  |  |  |  |  |  |
| <280 |  |  |  |  | 1.00 (Reference) |  |  |  |  |  | 1.00 (Reference) |
| ≥280 | 1.05 | 0.07 | 14.15 | <0.001 | 2.85 (2.47 ~ 3.30) |  | 0.57 | 0.08 | 7.18 | <0.001 | 1.76 (1.51 ~ 2.05) |
| NT-proBNP |  |  |  |  |  |  |  |  |  |  |  |
| <125 |  |  |  |  | 1.00 (Reference) |  |  |  |  |  |  |
| [125, 300) | -0.02 | 0.21 | -0.10 | 0.923 | 0.98 (0.65 ~ 1.48) |  |  |  |  |  |  |
| ≥300 | 0.51 | 0.15 | 3.39 | <0.001 | 1.67 (1.24 ~ 2.25) |  |  |  |  |  |  |
| FT_3_ |  |  |  |  |  |  |  |  |  |  |  |
| [3.1, 6.8] |  |  |  |  | 1.00 (Reference) |  |  |  |  |  | 1.00 (Reference) |
| <3.1 | 1.11 | 0.05 | 20.81 | <0.001 | 3.04 (2.74 ~ 3.38) |  | 0.81 | 0.06 | 14.25 | <0.001 | 2.24 (2.01 ~ 2.51) |
| >6.8 | -0.52 | 0.51 | -1.01 | 0.311 | 0.60 (0.22 ~ 1.62) |  | -0.49 | 0.52 | -0.96 | 0.338 | 0.61 (0.22 ~ 1.68) |
| LVEF |  |  |  |  |  |  |  |  |  |  |  |
| ≥50 |  |  |  |  | 1.00 (Reference) |  |  |  |  |  | 1.00 (Reference) |
| (40, 50) | -0.22 | 0.07 | -3.29 | 0.001 | 0.80 (0.70 ~ 0.91) |  | -0.24 | 0.07 | -3.40 | <0.001 | 0.78 (0.68 ~ 0.90) |
| ≤40 | -0.32 | 0.07 | -4.31 | <0.001 | 0.73 (0.63 ~ 0.84) |  | -0.26 | 0.08 | -3.42 | <0.001 | 0.77 (0.66 ~ 0.89) |

OR: Odds Ratio, CI: Confidence Interval, M:Male, F:Female.

**Supplementary Table 4.** Results of univariate and multivariate logistic regression analyses for cardiac death.

| Variables | univariate | | | | |  | multivariate | | | | |
| --- | --- | --- | --- | --- | --- | --- | --- | --- | --- | --- | --- |
|  | β | S.E | Z | *P* | OR (95%CI) |  | β | S.E | Z | *P* | OR (95%CI) |
| Age |  |  |  |  |  |  |  |  |  |  |  |
| [18, 45) |  |  |  |  | 1.00 (Reference) |  |  |  |  |  |  |
| [45, 65) | 0.08 | 0.14 | 0.55 | 0.579 | 1.08 (0.82 ~ 1.42) |  |  |  |  |  |  |
| [65, 75) | 0.13 | 0.15 | 0.88 | 0.376 | 1.14 (0.85 ~ 1.53) |  |  |  |  |  |  |
| [75, 90) | 0.29 | 0.15 | 1.89 | 0.059 | 1.33 (0.99 ~ 1.79) |  |  |  |  |  |  |
| ≥90 | -0.29 | 0.60 | -0.49 | 0.624 | 0.75 (0.23 ~ 2.40) |  |  |  |  |  |  |
| HR |  |  |  |  |  |  |  |  |  |  |  |
| [60, 100] |  |  |  |  | 1.00 (Reference) |  |  |  |  |  |  |
| <60 | 0.19 | 0.27 | 0.71 | 0.476 | 1.21 (0.72 ~ 2.04) |  |  |  |  |  |  |
| >100 | 0.64 | 0.10 | 6.57 | <0.001 | 1.90 (1.57 ~ 2.31) |  |  |  |  |  |  |
| PP |  |  |  |  |  |  |  |  |  |  |  |
| [30, 60] |  |  |  |  | 1.00 (Reference) |  |  |  |  |  | 1.00 (Reference) |
| <30 | 0.69 | 0.13 | 5.22 | <0.001 | 1.99 (1.54 ~ 2.57) |  | 0.59 | 0.14 | 4.37 | <0.001 | 1.80 (1.38 ~ 2.35) |
| >60 | -0.05 | 0.10 | -0.50 | 0.620 | 0.95 (0.77 ~ 1.16) |  | -0.03 | 0.11 | -0.30 | 0.767 | 0.97 (0.79 ~ 1.19) |
| BMI |  |  |  |  |  |  |  |  |  |  |  |
| [18.5, 24.0) |  |  |  |  | 1.00 (Reference) |  |  |  |  |  | 1.00 (Reference) |
| <18.5 | -1.13 | 0.36 | -3.12 | 0.002 | 0.32 (0.16 ~ 0.66) |  | -1.16 | 0.36 | -3.19 | 0.001 | 0.31 (0.15 ~ 0.64) |
| [24.0, 28.0) | -0.25 | 0.11 | -2.15 | 0.031 | 0.78 (0.62 ~ 0.98) |  | -0.18 | 0.12 | -1.58 | 0.114 | 0.83 (0.66 ~ 1.04) |
| ≥28.0 | 0.49 | 0.10 | 4.97 | <0.001 | 1.63 (1.35 ~ 1.98) |  | 0.53 | 0.10 | 5.33 | <0.001 | 1.71 (1.40 ~ 2.08) |
| WBC |  |  |  |  |  |  |  |  |  |  |  |
| [3.5, 9.5] |  |  |  |  | 1.00 (Reference) |  |  |  |  |  | 1.00 (Reference) |
| <3.5 | -0.22 | 0.34 | -0.66 | 0.511 | 0.80 (0.41 ~ 1.56) |  | -0.29 | 0.34 | -0.84 | 0.404 | 0.75 (0.38 ~ 1.47) |
| >9.5 | 0.89 | 0.09 | 10.32 | <0.001 | 2.45 (2.06 ~ 2.90) |  | 0.61 | 0.09 | 6.58 | <0.001 | 1.83 (1.53 ~ 2.20) |
| ALT |  |  |  |  |  |  |  |  |  |  |  |
| M: [9, 50], F: [7, 40] |  |  |  |  | 1.00 (Reference) |  |  |  |  |  |  |
| M: <9, F: <7 | 0.10 | 0.21 | 0.48 | 0.633 | 1.10 (0.74 ~ 1.65) |  |  |  |  |  |  |
| M: >50, F: >40 | 0.60 | 0.09 | 6.63 | <0.001 | 1.83 (1.53 ~ 2.19) |  |  |  |  |  |  |
| AST |  |  |  |  |  |  |  |  |  |  |  |
| M: [15, 40], F: [13, 35] |  |  |  |  | 1.00 (Reference) |  |  |  |  |  | 1.00 (Reference) |
| M: <15, F: <13 | 0.14 | 0.16 | 0.85 | 0.396 | 1.15 (0.83 ~ 1.59) |  | 0.15 | 0.17 | 0.93 | 0.351 | 1.17 (0.84 ~ 1.61) |
| M: >40, F: >35 | 0.65 | 0.09 | 7.41 | <0.001 | 1.92 (1.62 ~ 2.29) |  | 0.25 | 0.10 | 2.66 | 0.008 | 1.29 (1.07 ~ 1.55) |
| DBIL |  |  |  |  |  |  |  |  |  |  |  |
| ≤5 |  |  |  |  | 1.00 (Reference) |  |  |  |  |  | 1.00 (Reference) |
| >5 | 0.45 | 0.09 | 5.09 | <0.001 | 1.56 (1.32 ~ 1.86) |  | 0.33 | 0.09 | 3.62 | <0.001 | 1.39 (1.16 ~ 1.66) |
| ALB |  |  |  |  |  |  |  |  |  |  |  |
| [40, 55] |  |  |  |  | 1.00 (Reference) |  |  |  |  |  |  |
| <40 | 0.48 | 0.11 | 4.52 | <0.001 | 1.62 (1.31 ~ 1.99) |  |  |  |  |  |  |
| >55 | 0.33 | 0.37 | 0.89 | 0.373 | 1.39 (0.67 ~ 2.88) |  |  |  |  |  |  |
| Na^+^ |  |  |  |  |  |  |  |  |  |  |  |
| [137, 147] |  |  |  |  | 1.00 (Reference) |  |  |  |  |  | 1.00 (Reference) |
| <137 | 0.50 | 0.09 | 5.64 | <0.001 | 1.65 (1.39 ~ 1.97) |  | 0.18 | 0.09 | 1.96 | 0.050 | 1.20 (1.01 ~ 1.45) |
| >147 | 0.55 | 0.21 | 2.60 | 0.009 | 1.73 (1.14 ~ 2.62) |  | 0.25 | 0.22 | 1.14 | 0.255 | 1.28 (0.84 ~ 1.96) |
| Ca^2+^ |  |  |  |  |  |  |  |  |  |  |  |
| [2.11, 2.52] |  |  |  |  | 1.00 (Reference) |  |  |  |  |  |  |
| <2.11 | 0.22 | 0.09 | 2.47 | 0.013 | 1.24 (1.05 ~ 1.47) |  |  |  |  |  |  |
| >2.52 | 0.07 | 0.29 | 0.24 | 0.811 | 1.07 (0.61 ~ 1.88) |  |  |  |  |  |  |
| Glu |  |  |  |  |  |  |  |  |  |  |  |
| [3.9, 6.1] |  |  |  |  | 1.00 (Reference) |  |  |  |  |  | 1.00 (Reference) |
| <3.9 | 0.19 | 0.24 | 0.80 | 0.427 | 1.21 (0.76 ~ 1.93) |  | 0.13 | 0.24 | 0.52 | 0.600 | 1.13 (0.71 ~ 1.82) |
| >6.1 | 0.64 | 0.09 | 6.89 | <0.001 | 1.90 (1.58 ~ 2.28) |  | 0.41 | 0.10 | 4.30 | <0.001 | 1.51 (1.25 ~ 1.83) |
| CRP |  |  |  |  |  |  |  |  |  |  |  |
| ≤10 |  |  |  |  | 1.00 (Reference) |  |  |  |  |  |  |
| >10 | 0.27 | 0.12 | 2.20 | 0.028 | 1.31 (1.03 ~ 1.66) |  |  |  |  |  |  |
| D-dimer |  |  |  |  |  |  |  |  |  |  |  |
| <280 |  |  |  |  | 1.00 (Reference) |  |  |  |  |  | 1.00 (Reference) |
| ≥280 | 0.58 | 0.10 | 5.57 | <0.001 | 1.79 (1.46 ~ 2.20) |  | 0.21 | 0.11 | 1.91 | 0.056 | 1.24 (0.99 ~ 1.54) |
| NT-proBNP |  |  |  |  |  |  |  |  |  |  |  |
| <125 |  |  |  |  | 1.00 (Reference) |  |  |  |  |  | 1.00 (Reference) |
| [125, 300) | 0.02 | 0.40 | 0.05 | 0.962 | 1.02 (0.46 ~ 2.24) |  | -0.07 | 0.40 | -0.17 | 0.866 | 0.93 (0.42 ~ 2.06) |
| ≥300 | 0.90 | 0.29 | 3.06 | 0.002 | 2.46 (1.38 ~ 4.36) |  | 0.52 | 0.30 | 1.76 | 0.079 | 1.69 (0.94 ~ 3.03) |
| FT_3_ |  |  |  |  |  |  |  |  |  |  |  |
| [3.1, 6.8] |  |  |  |  | 1.00 (Reference) |  |  |  |  |  | 1.00 (Reference) |
| <3.1 | 0.84 | 0.09 | 9.90 | <0.001 | 2.32 (1.97 ~ 2.74) |  | 0.66 | 0.09 | 7.43 | <0.001 | 1.93 (1.62 ~ 2.30) |
| >6.8 | 0.11 | 0.59 | 0.18 | 0.857 | 1.11 (0.35 ~ 3.51) |  | 0.26 | 0.59 | 0.44 | 0.657 | 1.30 (0.41 ~ 4.14) |
| LVEF |  |  |  |  |  |  |  |  |  |  |  |
| ≥50 |  |  |  |  | 1.00 (Reference) |  |  |  |  |  | 1.00 (Reference) |
| (40, 50) | 0.27 | 0.10 | 2.66 | 0.008 | 1.31 (1.07 ~ 1.60) |  | 0.21 | 0.10 | 2.02 | 0.043 | 1.23 (1.01 ~ 1.51) |
| ≤40 | 0.19 | 0.11 | 1.72 | 0.085 | 1.21 (0.97 ~ 1.49) |  | 0.14 | 0.11 | 1.24 | 0.216 | 1.15 (0.92 ~ 1.43) |

OR: Odds Ratio, CI: Confidence Interval, M:Male, F:Female.

**Supplementary Table 5.** Results of multivariate logistic regression analyses for all-cause mortality in HFpEF, HFmrEF and HFrEF patients.

| Variables | HFpEF | |  | HFmrEF | |  | HFrEF | |
| --- | --- | --- | --- | --- | --- | --- | --- | --- |
|  | *P* | OR (95%CI) |  | *P* | OR (95%CI) |  | *P* | OR (95%CI) |
| Age |  |  |  |  |  |  |  |  |
| [18, 45) |  | 1.00 (Reference) |  |  | 1.00 (Reference) |  |  | 1.00 (Reference) |
| [45, 65) | 0.364 | 1.12 (0.87 ~ 1.44) |  | 0.216 | 1.32 (0.85 ~ 2.06) |  | 0.03 | 1.81 (1.06 ~ 3.10) |
| [65, 75) | 0.002 | 1.50 (1.16 ~ 1.93) |  | 0.055 | 1.58 (0.99 ~ 2.53) |  | 0.01 | 2.11 (1.20 ~ 3.71) |
| [75, 90) / ≥75 | <0.001 | 2.34 (1.82 ~ 3.01) |  | <0.001 | 2.72 (1.72 ~ 4.30) |  | <0.001 | 4.42 (2.54 ~ 7.70) |
| ≥90 / - | <0.001 | 4.04 (2.39 ~ 6.82) |  |  |  |  | 0.001 | 6.13 (2.01 ~ 18.67) |
| HR |  |  |  |  |  |  |  |  |
| [60, 100] |  |  |  |  | 1.00 (Reference) |  |  | 1.00 (Reference) |
| <60 |  |  |  | 0.997 | 1.00 (0.39 ~ 2.57) |  | 0.803 | 1.13 (0.44 ~ 2.93) |
| >100 |  |  |  | <0.001 | 1.68 (1.27 ~ 2.21) |  | 0.008 | 1.52 (1.11 ~ 2.06) |
| PP |  |  |  |  |  |  |  |  |
| [30, 60] |  | 1.00 (Reference) |  |  | 1.00 (Reference) |  |  |  |
| <30 | 0.009 | 1.43 (1.09 ~ 1.87) |  | 0.049 | 1.51 (1.01 ~ 2.26) |  |  |  |
| >60 | 0.673 | 0.97 (0.83 ~ 1.13) |  | 0.167 | 0.80 (0.59 ~ 1.10) |  |  |  |
| BMI |  |  |  |  |  |  |  |  |
| [18.5, 24.0) |  | 1.00 (Reference) |  |  | 1.00 (Reference) |  |  | 1.00 (Reference) |
| <18.5 | 0.004 | 0.57 (0.39 ~ 0.84) |  | 0.034 | 0.44 (0.21 ~ 0.94) |  | 0.048 | 0.44 (0.20 ~ 0.99) |
| [24.0, 28.0) | 0.004 | 0.77 (0.64 ~ 0.92) |  | 0.018 | 0.67 (0.48 ~ 0.93) |  | <0.001 | 0.51 (0.35 ~ 0.74) |
| ≥28.0 | <0.001 | 1.50 (1.28 ~ 1.76) |  | 0.002 | 1.56 (1.17 ~ 2.08) |  | 0.043 | 1.38 (1.01 ~ 1.90) |
| WBC |  |  |  |  |  |  |  |  |
| [3.5, 9.5] |  | 1.00 (Reference) |  |  | 1.00 (Reference) |  |  | 1.00 (Reference) |
| <3.5 | <0.001 | 1.91 (1.37 ~ 2.66) |  | 0.012 | 2.38 (1.21 ~ 4.66) |  | 0.064 | 2.24 (0.95 ~ 5.27) |
| >9.5 | <0.001 | 1.81 (1.56 ~ 2.09) |  | <0.001 | 2.38 (1.84 ~ 3.08) |  | <0.001 | 2.86 (2.15 ~ 3.82) |
| ALT |  |  |  |  |  |  |  |  |
| M: [9, 50], F: [7, 40] |  |  |  |  |  |  |  |  |
| M: <9, F: <7 |  |  |  |  |  |  |  |  |
| M: >50, F: >40 |  |  |  |  |  |  |  |  |
| AST |  |  |  |  |  |  |  |  |
| M: [15, 40], F: [13, 35] |  | 1.00 (Reference) |  |  |  |  |  |  |
| M: <15, F: <13 | 0.297 | 1.14 (0.89 ~ 1.46) |  |  |  |  |  |  |
| M: >40, F: >35 | 0.018 | 1.20 (1.03 ~ 1.40) |  |  |  |  |  |  |
| DBIL |  |  |  |  |  |  |  |  |
| ≤5 |  | 1.00 (Reference) |  |  | 1.00 (Reference) |  |  | 1.00 (Reference) |
| >5 | <0.001 | 1.47 (1.27 ~ 1.70) |  | 0.017 | 1.37 (1.06 ~ 1.78) |  | 0.133 | 1.24 (0.94 ~ 1.64) |
| ALB |  |  |  |  |  |  |  |  |
| [40, 55] |  |  |  |  |  |  |  | 1.00 (Reference) |
| <40 |  |  |  |  |  |  | 0.014 | 1.67 (1.11 ~ 2.51) |
| >55 |  |  |  |  |  |  | 0.221 | 2.04 (0.65 ~ 6.38) |
| Na^+^ |  |  |  |  |  |  |  |  |
| [137, 147] |  | 1.00 (Reference) |  |  |  |  |  | 1.00 (Reference) |
| <137 | 0.059 | 1.15 (0.99 ~ 1.34) |  |  |  |  | 0.059 | 1.33 (0.99 ~ 1.79) |
| >147 | 0.013 | 1.55 (1.10 ~ 2.18) |  |  |  |  | 0.001 | 2.62 (1.46 ~ 4.71) |
| Ca^2+^ |  |  |  |  |  |  |  |  |
| [2.11, 2.52] |  |  |  |  |  |  |  |  |
| <2.11 |  |  |  |  |  |  |  |  |
| >2.52 |  |  |  |  |  |  |  |  |
| Glu |  |  |  |  |  |  |  |  |
| [3.9, 6.1] |  | 1.00 (Reference) |  |  | 1.00 (Reference) |  |  | 1.00 (Reference) |
| <3.9 | 0.84 | 1.04 (0.72 ~ 1.51) |  | 0.203 | 1.55 (0.79 ~ 3.06) |  | 0.174 | 1.72 (0.79 ~ 3.74) |
| >6.1 | <0.001 | 1.47 (1.27 ~ 1.71) |  | <0.001 | 1.85 (1.39 ~ 2.47) |  | 0.034 | 1.39 (1.03 ~ 1.89) |
| CRP |  |  |  |  |  |  |  |  |
| ≤10 |  | 1.00 (Reference) |  |  |  |  |  |  |
| >10 | 0.099 | 0.85 (0.70 ~ 1.03) |  |  |  |  |  |  |
| D-dimer |  |  |  |  |  |  |  |  |
| <280 |  | 1.00 (Reference) |  |  | 1.00 (Reference) |  |  | 1.00 (Reference) |
| ≥280 | <0.001 | 1.95 (1.62 ~ 2.36) |  | 0.004 | 1.66 (1.18 ~ 2.34) |  | 0.033 | 1.54 (1.04 ~ 2.28) |
| NT-proBNP |  |  |  |  |  |  |  |  |
| <125 |  |  |  |  |  |  |  |  |
| [125, 300) |  |  |  |  |  |  |  |  |
| ≥300 |  |  |  |  |  |  |  |  |
| FT_3_ |  |  |  |  |  |  |  |  |
| [3.1, 6.8] |  | 1.00 (Reference) |  |  | 1.00 (Reference) |  |  | 1.00 (Reference) |
| <3.1 | <0.001 | 2.15 (1.87 ~ 2.46) |  | <0.001 | 2.52 (1.95 ~ 3.24) |  | <0.001 | 2.56 (1.93 ~ 3.39) |
| >6.8 | 0.147 | 0.23 (0.03 ~ 1.67) |  | 0.584 | 1.51 (0.34 ~ 6.68) |  | 0.978 | 0.97 (0.12 ~ 8.04) |

OR: Odds Ratio, CI: Confidence Interval, M:Male, F:Female.

**Supplementary Table 6.** Results of multivariate logistic regression analyses for cardiac death in HFpEF, HFmrEF and HFrEF patients.

| Variables | HFpEF | |  | HFmrEF | |  | HFrEF | |
| --- | --- | --- | --- | --- | --- | --- | --- | --- |
|  | *P* | OR (95%CI) |  | *P* | OR (95%CI) |  | *P* | OR (95%CI) |
| Age |  |  |  |  |  |  |  |  |
| [18, 45) |  |  |  |  |  |  |  |  |
| [45, 65) |  |  |  |  |  |  |  |  |
| [65, 75) |  |  |  |  |  |  |  |  |
| [75, 90) |  |  |  |  |  |  |  |  |
| ≥90 |  |  |  |  |  |  |  |  |
| HR |  |  |  |  |  |  |  |  |
| [60, 100] |  |  |  |  | 1.00 (Reference) |  |  |  |
| <60 |  |  |  | 0.711 | 0.76 (0.18 ~ 3.21) |  |  |  |
| >100 |  |  |  | 0.031 | 1.52 (1.04 ~ 2.22) |  |  |  |
| PP |  |  |  |  |  |  |  |  |
| [30, 60] |  | 1.00 (Reference) |  |  | 1.00 (Reference) |  |  |  |
| <30 | 0.021 | 1.61 (1.07 ~ 2.41) |  | <0.001 | 2.30 (1.44 ~ 3.67) |  |  |  |
| >60 | 0.902 | 0.98 (0.75 ~ 1.29) |  | 0.329 | 0.80 (0.51 ~ 1.25) |  |  |  |
| BMI |  |  |  |  |  |  |  |  |
| [18.5, 24.0) |  | 1.00 (Reference) |  |  | 1.00 (Reference) |  |  | 1.00 (Reference) |
| <18.5 | 0.023 | 0.31 (0.11 ~ 0.85) |  | 0.107 | 0.31 (0.07 ~ 1.29) |  | 0.098 | 0.30 (0.07 ~ 1.25) |
| [24.0, 28.0) | 0.532 | 0.91 (0.67 ~ 1.23) |  | 0.485 | 0.85 (0.53 ~ 1.35) |  | 0.081 | 0.65 (0.40 ~ 1.06) |
| ≥28.0 | <0.001 | 1.74 (1.33 ~ 2.28) |  | <0.001 | 1.99 (1.33 ~ 2.97) |  | 0.346 | 1.23 (0.80 ~ 1.88) |
| WBC |  |  |  |  |  |  |  |  |
| [3.5, 9.5] |  | 1.00 (Reference) |  |  | 1.00 (Reference) |  |  | 1.00 (Reference) |
| <3.5 | 0.299 | 0.62 (0.25 ~ 1.53) |  | 0.391 | 0.42 (0.06 ~ 3.07) |  | 0.172 | 2.33 (0.69 ~ 7.80) |
| >9.5 | 0.004 | 1.44 (1.12 ~ 1.85) |  | <0.001 | 2.09 (1.46 ~ 2.99) |  | <0.001 | 2.98 (2.04 ~ 4.35) |
| ALT |  |  |  |  |  |  |  |  |
| M: [9, 50], F: [7, 40] |  | 1.00 (Reference) |  |  |  |  |  |  |
| M: <9, F: <7 | 0.696 | 1.11 (0.65 ~ 1.90) |  |  |  |  |  |  |
| M: >50, F: >40 | 0.002 | 1.51 (1.16 ~ 1.96) |  |  |  |  |  |  |
| AST |  |  |  |  |  |  |  |  |
| M: [15, 40], F: [13, 35] |  |  |  |  | 1.00 (Reference) |  |  |  |
| M: <15, F: <13 |  |  |  | 0.247 | 1.48 (0.76 ~ 2.87) |  |  |  |
| M: >40, F: >35 |  |  |  | 0.032 | 1.51 (1.04 ~ 2.20) |  |  |  |
| DBIL |  |  |  |  |  |  |  |  |
| ≤5 |  | 1.00 (Reference) |  |  |  |  |  |  |
| >5 | <0.001 | 1.70 (1.33 ~ 2.16) |  |  |  |  |  |  |
| ALB |  |  |  |  |  |  |  |  |
| [40, 55] |  |  |  |  |  |  |  | 1.00 (Reference) |
| <40 |  |  |  |  |  |  | 0.025 | 1.85 (1.08 ~ 3.17) |
| >55 |  |  |  |  |  |  | 0.951 | 1.07 (0.14 ~ 8.34) |
| Na^+^ |  |  |  |  |  |  |  |  |
| [137, 147] |  |  |  |  |  |  |  | 1.00 (Reference) |
| <137 |  |  |  |  |  |  | 0.064 | 1.46 (0.98 ~ 2.17) |
| >147 |  |  |  |  |  |  | 0.004 | 2.99 (1.41 ~ 6.34) |
| Ca^2+^ |  |  |  |  |  |  |  |  |
| [2.11, 2.52] |  |  |  |  |  |  |  |  |
| <2.11 |  |  |  |  |  |  |  |  |
| >2.52 |  |  |  |  |  |  |  |  |
| Glu |  |  |  |  |  |  |  |  |
| [3.9, 6.1] |  | 1.00 (Reference) |  |  | 1.00 (Reference) |  |  | 1.00 (Reference) |
| <3.9 | 0.673 | 1.14 (0.62 ~ 2.10) |  | 0.924 | 1.05 (0.37 ~ 3.03) |  | 0.403 | 1.57 (0.54 ~ 4.55) |
| >6.1 | 0.002 | 1.49 (1.16 ~ 1.92) |  | 0.011 | 1.67 (1.13 ~ 2.49) |  | 0.042 | 1.54 (1.02 ~ 2.33) |
| CRP |  |  |  |  |  |  |  |  |
| ≤10 |  |  |  |  |  |  |  |  |
| >10 |  |  |  |  |  |  |  |  |
| D-dimer |  |  |  |  |  |  |  |  |
| <280 |  | 1.00 (Reference) |  |  |  |  |  |  |
| ≥280 | 0.128 | 1.25 (0.94 ~ 1.67) |  |  |  |  |  |  |
| NT-proBNP |  |  |  |  |  |  |  |  |
| <125 |  | 1.00 (Reference) |  |  |  |  |  |  |
| [125, 300) | 0.851 | 1.09 (0.44 ~ 2.71) |  |  |  |  |  |  |
| ≥300 | 0.105 | 1.75 (0.89 ~ 3.45) |  |  |  |  |  |  |
| FT_3_ |  |  |  |  |  |  |  |  |
| [3.1, 6.8] |  | 1.00 (Reference) |  |  | 1.00 (Reference) |  |  | 1.00 (Reference) |
| <3.1 | <0.001 | 1.56 (1.23 ~ 1.97) |  | <0.001 | 2.59 (1.83 ~ 3.66) |  | <0.001 | 2.34 (1.60 ~ 3.43) |
| >6.8 | 0.740 | 0.71 (0.10 ~ 5.19) |  | 0.554 | 1.84 (0.24 ~ 13.87) |  | 0.583 | 1.81 (0.22 ~ 14.91) |

**
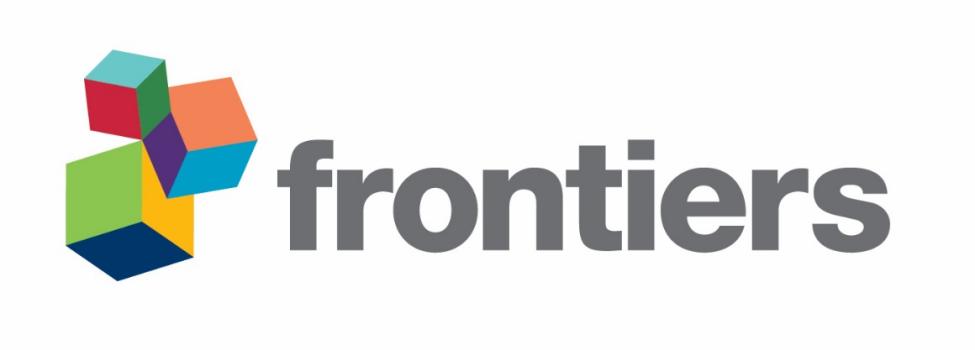
**

**Figure legends**

**Supplementary Figure 1.** The top 15 feature importance scores generated by the random forest model.
